# The red distribution width and the platelet distribution width as prognostic predictors in gastric cancer

**DOI:** 10.1186/s12876-017-0685-7

**Published:** 2017-12-20

**Authors:** Shiqing Cheng, Fuyan Han, Yong Wang, Yanqiu Xu, Teng Qu, Ying Ju, Zhiming Lu

**Affiliations:** Department of Laboratory Medicine, Shandong Provincial Hospital Affiliated to Shandong University, Jinan, Shandong 250021 China

**Keywords:** Red distribution width, Platelet distribution width, Gastric cancer, Early stage gastric cancer

## Abstract

**Background:**

Increasing attention is focused on the relationship of inflammation biomarkers with malignant tumors. The purpose of the present study was to detect whether the preoperative the red distribution width (RDW) and the platelet distribution width (PDW) can be used to distinguish patients with gastric cancer (GC) or early stage GC from the healthy controls and predict the progression and prognosis of the GC.

**Methods:**

The RDW and PDW values of 227 patients with GC and 164 patients with early GC were retrospectively analyzed comparing with 101 healthy controls. In addition, the clinicopathological features, survival curves and prognosis of the patients with GC were compared between the high and low groups according to the RDW and PDW values.

**Results:**

Significant higher RDW and lower PDW were detected in patients with GC and early GC compared to the healthy controls. A higher RDW was significantly associated with older age, a larger tumor diameter, deeper tumor infiltration, and lymph node metastasis while a lower PDW was significantly associated with male, older age, a larger tumor diameter, deeper tumor infiltration, elevated CEA and CA125. Increased RDW was significantly associated with worse overall survival (OS) and disease-free survival (DFS) for GC (*P* = 0.042 and *P* = 0.033, respectively) and early GC (*P* = 0.037 and *P* = 0.009, respectively) while decreased PDW indicated a significantly association with poor DFS for early GC (*P* = 0.006). Univariate and multivariate survival analysis showed that RDW and PDW can act as independent prognostic factors for DFS (*P* = 0.028 and *P* = 0.020) in patients with early GC.

**Conclusion:**

The preoperative RDW and PDW were simple and convenient predictive factors for the progression and prognosis of patients with GC.

## Background

Gastric cancer (GC) is still one of the most common causes of cancer-related death despite improvements in treatment modalities worldwide [1]. The incidence rate of gastric cancer varies widely in different areas and is particularly common in East Asia [2]. Most patients are diagnosed at the advanced stage and have either regional or distant metastasis with the 5-year survival less than 10% [3]. Therefore, it is important to identify prognostic factors for these patients in order to select appropriate treatment for patients. Both genetic and environmental factors are related to GC development [4]. Previous studies have verified the relationship between inflammation and progress of gastric carcinoma [5–7]. It is well known that cancer can induce malnutrition and chronic inflammatory response, and cancer-related inflammation is a critical factor for progression and prognosis of many cancers [7, 8]. In recent years, there is a growing interest in establishing novel non-invasive predictive biomarkers from hematological and serologic parameters for various inflammatory diseases and cancers. Although complete blood count (CBC) have been routinely available to clinicians, the roles of several parameters in the diagnosis and management of patients with malignancies, such as RDW and PDW, remain obscure.

Red blood cell distribution width (RDW) is widely used laboratory parameter for anemia [9]. However, recent studies have reported that RDW can be used laboratory parameter for inflammatory diseases and cancers, such as atherosclerosis, inflammatory bowel diseases, breast cancer and lung cancer [10–13]. Platelet distribution width (PDW) is a measure of variation in platelet size and a direct flow cytometric measurement of platelet cell volume. PDW has been evaluated as a marker of platelet morphology and activation [14, 15]. Recent studies also showed the association of PDW with CBC and CRP which indicated the wide relation between platelets and inflammation [16].

However, to the best of our knowledge, there were few specific studies comprehensively evaluating the values of RDW and PDW indices in GC patients. We, therefore, aimed to determine whether the two parameters could be indicators used for assessment of disease diagnosis and prognosis by retrospectively analyzing the correlation between the values of RDW, PDW and the clinical data of GC patients.

## Methods

### Subjects

All clinicopathological data was analyzed retrospectively in 227 patients with GC undergoing radical surgery resection and 101 healthy volunteers as controls which were recruited in Shandong Provincial Hospital Affiliated to Shandong University from July 2010 to December 2014. Furthermore, we collected 164 patients with early stage (T1 stage) GC for comparative analysis with the healthy controls. All patients with histopathologically diagnosed as gastric cancer by two pathologists after a radical resection were selected. Patients were selected for the present study according to the following inclusion criteria: confirmed histopathologic diagnosis; complete whole blood count before surgery; clinicopathological and follow-up data.

The patients with clinical signs of infection, hematologic disease, anaemia, liver disease, with blood transfusion made in the last three months, venous thrombosis detected in the last six months, severe coronary heart disease, autoimmune disease and a history of other malignancies were excluded from the study. A total of 42 patients were excluded due to the criteria in question in our study. Clinicopathological parameters were obtained from medical records. All patients were regularly followed up by telephone interviews and the last follow-up assessment was conducted in November 2016. All patients were staged according to the criteria of American Joint Committee on Cancer Staging (7th edition) [17]. As the primary study end point, overall survival (OS)was calculated from the date of surgery to death from any cause. The secondary end point was disease-free survival (DFS), which was defined as the time from surgery to identification of disease recurrence, either radiological or histological. Approval for the study was granted by the Ethics Committee of the Shandong Provincial Hospital Affiliated to Shandong University with written informed consents from all participants.

### Blood sampling

RDW and PDW values of patients were detected one week before radical surgery with an automated hematology analyzer XE-2100 (Sysmex, Kobe, Japan). Tumor biomarkers such as carcinoembryonic antigen (CEA) and carbohydrate antigen 199 (CA199) were measured within one month before surgery using a Cobas e601 analyzer (Roche Diagnostics, Mannheim, Germany). We calculated the median values of RDW and PDW in all 227 patients and 164 patients at early stage respectively (13 and 11.5% in all 227 patients, 12.85 and 11.95% in 164 patients at early stage). These median values were used as cutoff values of RDW and PDW to classify patients as high and low groups.

### Statistical analysis

Statistical analysis was performed by using SPSS statistical soft ware version 19.0 (SPSS Inc., Chicago, USA). The normal distributions of continuous variables were shown as mean ± SD while the non-normal distributions of continuous variables were presented as median(interquartile range). Categorical variables were shown as frequencies. While the mean differences between groups were compared by Student’s t test, otherwise, Mann Whitney U test was applied for comparisons of the median values. Differences between categories of each clinicopathological feature were analyzed using the chi-squared test. ROC curves were used to determine the diagnostic value of the RDW and PDW. We used the Kaplan-Meier analysis to calculate the OS and DFS rates and the log-rank test to compare the survival rate curves. Significant parameters for survival in univariate analysis were introduced into multivariate Cox proportional hazards model to determine independent prognostic factors. *P* value < 0.05 was considered statistical significance.

## Results

### Comparison of RDW and PDW values between patients with GC and healthy controls

**Fig. 1:**
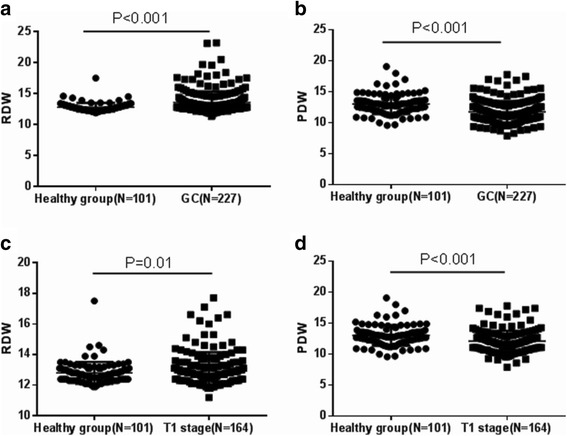
Comparison of RDW and PDW values between patients with GC and healthy controls. **a** RDW values between patients with GC and healthy controls. **b** PDW values between patients with GC and healthy controls. **c** RDW values between patients with early stage GC and healthy controls. **d** PDW values between patients with early stage GC and healthy controls

The mean ± SD of preoperative RDW in 227 GC patients and healthy controls were (13.57 ± 1.804)% and (12.80 ± 0.712)%, respectively. The difference was significant (*t* = 4.137, *P* < 0.001, Fig. 1a). Likewise, a significant difference existed in PDW value. The mean ± SD of preoperative PDW in patients was (11.80 ± 1.733)% which was significantly lower than (13.03 ± 1.619)% in healthy controls (*t* = 6.019, *P* < 0.001, Fig. 1b). We also calculated the mean ± SD of RDW and PDW in 164 patients with early stage GC, which were (13.11 ± 1.050)% and (12.11 ± 1.698)%, respectively and there were significant differences comparing to healthy controls (*t* = 2.578, *P* = 0.010 and *t* = 4.337, *P* < 0.001, respectively, Fig. 1c, d).

### ROC curves analysis results

**Fig. 2:**
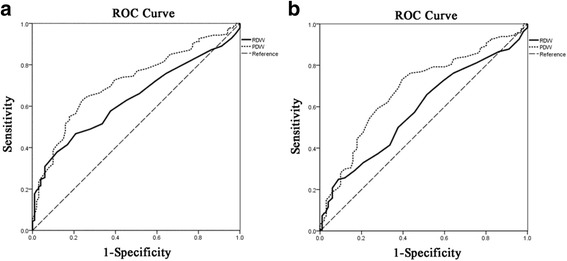
The results of ROC curve analysis for the predictive power of RDW and PDW in predicting presence of GC and early stage GC. **a** ROC curve of RDW and PDW in predicting presence of GC. **b** ROC curve of RDW and PDW in predicting presence of early stage GC

We used ROC curve analysis to verify the predictive power of RDW and PDW in predicting presence of GC and early stage GC. For GC, the AUC of RDW was 0.635 (95%CI = 0.575–0.695, *P* < 0.001) and the AUC of PDW was 0.715 (95%CI = 0.679–0.785, *P* < 0.001) for predicting the presence of GC (Fig. 2a). While for early stage GC, the AUC of RDW and PDW was 0.585 (95%CI = 0.516–0.654, *P* = 0.020) and 0.683 (95%CI = 0.618–0.749, *P* < 0.001), respectively, for predicting the presence of early stage GC (Fig. 2b).

### Relationships between RDW, PDW and clinicopathological characteristics

The differences of clinicopathological characteristics between the two groups in 227 GC patients according to RDW and PDW were exhibited in Table 1. A higher RDW was significantly associated with older age, a larger tumor diameter, deeper tumor infiltration, and Lymph node metastasis (*P* < 0.001, *P* = 0.001, *P* = 0.008 and *P* = 0.014, respectively). A lower PDW was significantly associated with male, older age, a larger tumor diameter, deeper tumor infiltration, elevated CEA and CA125 (*P* = 0.039, *P* = 0.007, *P* = 0.005, *P* = 0.047, *P* = 0.004 and *P* = 0.041, respectively). The differences of clinicopathological characteristics between the two groups according to RDW and PDW in 164 patients with early stage GC were showed in Table 2. A high RDW was only significantly associated with older age. However, no prominent difference existed in other features.

**Table 1:** Comparison of clinicopathological parameters of 227 patients with GC between high and low groups in terms of RDW and PDW

Characteristics	Cases (n)	RDW < 13 n(%)	RDW ≥ 13 n(%)	*P* value^a^	PDW < 11.5 n(%)	PDW ≥ 11.5 n(%)	*P* Value^a^
Gender				0.467			0.039
Male	176	83(75.5)	93(79.5)		91(83.5)	85(72.0)	
Female	51	27(24.5)	24(20.5)		18(16.5)	33(28.0)	
Age(years)				< 0.001			0.007
< 60	119	76(69.1)	43(36.8)		47(43.1)	72(61.0)	
≥ 60	108	34(30.9)	74(63.2)		62(56.9)	46(39.0)	
Tumor diameter (cm)				0.001			0.005
≤ 4	101	61(55.5)	40(34.2)		38(34.9)	63(53.4)	
> 4	116	49(44.5)	77(65.8)		71(65.1)	55(46.6)	
Differentiation				0.569			0.785
Well	7	3(2.7)	4(3.4)		3(2.8)	4(3.4)	
Moderate	71	31(28.2)	40(34.2)		32(29.4)	39(33.1)	
Poor	149	76(69.1)	73(62.4)		74(67.9)	75(63.6)	
Depth of tumor				0.008			0.047
T1 + T2	64	40(36.4)	24(20.5)		24(22.0)	40(33.9)	
T3 + T4	163	70(63.6)	93(79.5)		85(78.0)	78(66.1)	
Lymph node metastasis				0.014			0.085
N0	73	44(40.0)	29(24.8)		29(26.6)	44(37.3)	
N1 + N2 + N3	154	66(60.0)	88(75.2)		80(73.4)	74(62.7)	
Distance metastasis				0.257			0.417
M0	194	91(82.7)	103(88.0)		91(83.5)	103(87.3)	
M1	33	19(17.3)	14(12.0)		18(16.5)	15(12.7)	
pStage				0.111			0.131
I + II	83	46(41.8)	37(31.6)		36(33.1)	47(39.8)	
III + IV	144	64(58.2)	80(68.4)		73(66.9)	71(60.2)	
CEA(ng/ml)				0.250			0.004
≤ 5	188	92(87.6)	96(82.1)		83(78.3)	105(92.1)	
> 5	34	13(12.4)	21(17.9)		23(21.7)	9(7.9)	
CA125(U/ml)				0.553			0.041
≤ 35	181	85(94.4)	96(92.3)		86(89.6)	95(96.9)	
> 35	13	5(5.6)	8(7.7)		10(10.4)	3(3.1)	
CA199(U/ml)				0.563			0.637
≤ 39	183	84(85.7)	99(88.4)		86(86.0)	97(88.2)	
> 39	27	14(14.3)	13(11.6)		14(14.0)	13(11.8)	

*Abbreviations*: *RDW* red blood cell distribution width, *PDW* platelet distribution width, *CEA* carcinoembryonic antigen, *CA125* carbohydrate antigen 125, *CA199* carbohydrate antigen 199

^a^Value was calculated by χ^2^ test

**Table 2:** Comparison of clinicopathological characteristics of 164 patients with GC at early stage between high and low groups in terms of RDW and PDW

Characteristics	Cases (N)	RDW < 12.85 n(%)	RDW ≥ 12.85 n(%)	*P* value^a^	PDW < 11.95 n(%)	PDW ≥ 11.95 n(%)	*P* Value^a^
Gender				1.000			0.711
Male	126	63(76.8)	63(76.8)		64(78.0)	62(75.6)	
Female	38	19(23.2)	19(23.2)		18(22.0)	20(24.4)	
Age(years)				0.008			0.271
< 60	73	45(54.9)	28(34.1)		33(40.2)	40(48.8)	
≥ 60	91	37(45.1)	54(65.9)		49(59.8)	42(51.2)	
Tumor diameter (cm)				0.732			0.732
≤ 4	155	77(93.9)	78(95.1)		77(93.9)	78(95.1)	
> 4	9	5(6.1)	4(4.9)		5(6.1)	4(4.9)	
Differentiation				0.437			0.537
Well	21	12(14.6)	9(11.0)		11(13.4)	10(12.2)	
Moderate	74	33(40.2)	41(50.0)		40(48.8)	34(41.5)	
Poor	69	37(45.1)	32(39.0)		31(37.8)	38(46.3)	
Lymph node metastasis				0.392			0.200
N0	138	67(81.7)	71(86.6)		72(87.8)	66(80.5)	
N1 + N2 + N3	26	15(18.3)	11(13.4)		10(12.2)	16(19.5)	
Distance metastasis				0.053			0.246
M0	157	76(92.7)	81(98.8)		80(97.6)	77(93.9)	
M1	7	6(7.3)	1(1.2)		2(2.4)	5(6.1)	
pStage				0.053			0.246
I + II	157	76(92.7)	81(98.8)		76(92.7)	70(85.4)	
III + IV	7	6 (7.3)	1(1.2)		6(7.3)	12(14.6)	
CEA(ng/ml)				0.110			0.384
≤ 5	134	67(93.1)	67(98.5)		65(94.2)	69(97.2)	
> 5	6	5(6.9)	1(1.5)		4(5.8)	2(2.8)	
CA125(U/ml)				0.955			0.504
≤ 35	75	36(97.3)	39(97.5)		33(100.0)	42(95.5)	
> 35	2	1(2.7)	1(2.5)		0 (0.0)	2(4.5)	
CA199(U/ml)				0.563			0.117
≤ 39	183	84(85.7)	99(88.4)		64(95.5)	69(100.0)	
> 39	27	14(14.3)	13(11.6)		3(4.5)	0(0.0)	

*Abbreviations*: *RDW* red blood cell distribution width, *PDW* platelet distribution width, *CEA* carcinoembryonic antigen, *CA125* carbohydrate antigen 125, *CA199* carbohydrate antigen 199

^a^Value was calculated by χ^2^ test

### Associations of RDW and PDW with survival of GC

**Fig. 3:**
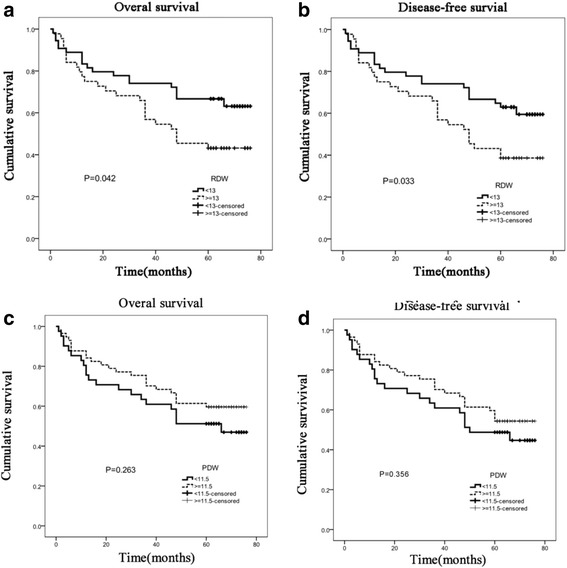
Kaplan-Meier survival curves stratified by RDW and PDW in 227 patients with GC. **a** Kaplan-Meier curves for OS by RDW in all GC, *P* = 0.042. **b** Kaplan-Meier curves for DFS by RDW in all GC, *P* = 0.033. **c** Kaplan-Meier curves for OS by PDW in all GC. **d** Kaplan-Meier curves for DFS by PDW in all GC. *P* values were determined using the log-rank test

**Fig. 4:**
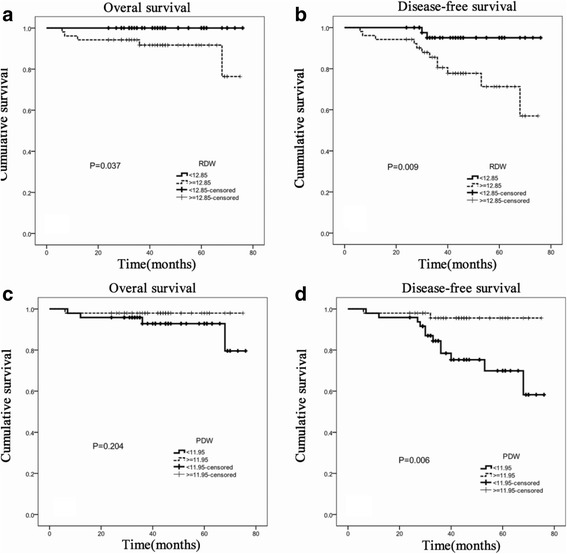
Kaplan-Meier survival curves stratified by RDW and PDW in 164 patients with GC at early stage. **a** Kaplan-Meier curves for OS by RDW in early stage GC. **b** Kaplan-Meier curves for DFS by RDW in early stage GC. **c** Kaplan-Meier curves for OS by PDW in early stage GC. **d** Kaplan-Meier curves for DFS by PDW in early stage GC. P values were determined using the log-rank test

To investigate whether the RDW and PDW were associated with survival of GC, we performed Kaplan-Meier curves for OS and DFS for all GC (Fig. 3) and early stage GC (Fig. 4). For the 227 patients with GC, the median follow-up duration was 61 months with a range of 1 to 76 months and for the 164 patients with early stage GC, the median follow-up duration was 45 months with a range of 6 to 76 months. A high RDW showed a worse OS (*P* = 0.042) and a shorter DFS (*P* = 0.033) in 227 GC while PDW had no significant association with OS and DFS (*P* = 0.263 and *P* = 0.356, respectively). For 164 early stage GC, significantly worse OS (*P* = 0.037) and DFS (*P* = 0.009) were also seen in patients with high RDW group than those with low RDW group. Furthermore, the low PDW group exhibited a poorer DFS than the high group (*P* = 0.006).

### Associations of RDW and PDW with prognosis of GC

To verify whether the RDW and PDW were the independent prognosis factors of GC, univariate and multivariate survival analysis for OS and DFS was performed. The analysis for 227 GC was exhibited in Table 3, a univariate analysis showed that tumor diameter (*P* = 0.001), degree of differentiation (*P* = 0.041), depth of tumor (*P* < 0.001), lymph node metastasis (*P* < 0.001), distant metastasis (*P* = 0.026), pStage (*P* < 0.001) and CA199 (*P* = 0.017) were significantly associated with OS. Moreover, a similar association with respect to DFS was also observed for the RDW (*P* = 0.044), age (*P* = 0.044), tumor diameter (*P* = 0.001), depth of tumor (*P* < 0.001), lymph node metastasis (*P* < 0.001), pStage (*P* < 0.001) and CA199 (*P* = 0.009). All significant prognostic factors tested by univariate analysis were evaluated by multivariate analysis. Depth of tumor and pStage were independent risk factors of OS (*P* = 0.006 and *P* = 0.004) and DFS (*P* = 0.010 and *P* = 0.005) in GC. For 164 patients with early stage GC, univariate and multivariate survival analysis showed that RDW and PDW can act as independent prognostic factors for DFS (*P* = 0.028 and *P* = 0.020), but no significant factor was found for OS (Table 4).

**Table 3:** Univariate and multivariate survival analysis results of 227 patients with GC

Clinicopathological feature	Univariate^a^(OS)		Multivariate^b^(OS)		Univariate^a^(DFS)		Multivariate^b^(DFS)	
	HR (95% CI)	*P* value	HR(95% CI)	*P* value	HR (95% CI)	*P* value	HR(95% CI)	*P* value
RDW(<13/≥13)	1.801 (0.985–3.291)	0.056			1.807 (1.015–3.217)	0.044		
PDW(<11.5/≥11.5)	0.701 (0.385–1.275)	0.244			0.754 (0.425–1.337)	0.333		
Gender(Male/Female)	0.764 (0.355–1.648)	0.493			0.908 (0.452–1.827)	0.787		
Age(<60/≥60)	1.541 (0.844–2.814)	0.160			1.535 (0.863–2.730)	0.044		
Tumor diameter(≤4/>4)	2.890 (1.553–5.375)	0.001			2.633 (1.465–4.731)	0.001		
Differentiation (Well/Moderate/ Poor)	1.968 (1.027–3.772)	0.041			1.650 (0.926–2.941)	0.089		
Depth of tumor (T1 + T2/T3 + T4)	2.169 (1.613–2.916)	< 0.001	1.722(1.170–2.535)	0.006	2.097 (1.569–2.802)	< 0.001	1.655(1.130–2.422)	0.010
Lymph node metastasis (N0/N1 + N2 + N3)	5.548 (2.335–13.181)	< 0.001			4.548 (2.118–9.763)	< 0.001		
Distance metastasis (M0/M1)	2.403 (1.112–5.193)	0.026			2.137 (0.996–4.584)	0.051		
pStage(I + II/III + IV)	7.841 (3.075–19.994)	< 0.001	4.311(1.591–11.682)	0.004	6.179 (2.755–13.858)	< 0.001	3.517(1.470–8.413)	0.005
CEA(≤5/>5)	1.240 (0.552–2.788)	0.602			1.116 (0.500–2.492)	0.789		
CA125(≤35/>35)	2.606 (0.353–19.240)	0.348			2.606 (0.353–19.240)	0.348		
CA199(≤39/>39)	2.405 (1.173–4.930)	0.017			2.478 (1.249–4.917)	0.009		

*Abbreviations*: *OS* overall survival, *DFS* disease-free survival, *HR* hazard ratio, *CI* confidence interval, *RDW* red blood cell distribution width, *CEA* carcinoembryonic antigen, *CA125* carbohydrate antigen 125, *CA199* carbohydrate antigen 199

^a^Performed using the Kaplan–Meier analysis model and the log-rank test; values of *P* < 0.05 in the univariate analysis were entered into a multivariate analysis

^b^performed using Cox proportional hazards models with the forward likelihood method

**Table 4:** Univariate and multivariate survival analysis results of 164 patients with GC at early stage

Variables	Univariate^a^ (OS)		Univariate^a^ (DFS)		Multivariate^b^(DFS)	
	HR(95% CI)	*P* value	HR(95% CI)	*P* value	HR(95% CI)	*P* value
RDW(<12.85/≥12.85)	57.198(0.042–7.805E4)	0.272	5.776(1.292–25.821)	0.022	5.346(1.195–23.917)	0.028
PDW(<11.95/≥11.95)	0.265 (0.029–2.387)	0.236	0.158(0.035–0.707)	0.016	0.170(0.038–0.761)	0.020
Gender(Male/Female)	1.526 (0.158–14.733)	0.715	2.983(0.973–9.150)	0.056		
Age(<60/≥60)	2.264 (0.242–21.168)	0.474	1.145(0.378–3.470)	0.811		
Tumor diameter(≤4/>4)	0.046 (0.000–1.144E7)	0.754	2.507(0.318–19.758)	0.383		
Differentiation (Well/Moderate/ Poor)	2.384 (0.487–11.664)	0.284	0.717(0.334–1.540)	0.394		
Lymph node metastasis (N0/N1 + N2 + N3)	1.460 (0.201–10.620)	0.708	1.706(0.513–5.671)	0.383		
Distance metastasis (M0/M1)	0.047(0.000–1.212E9)	0.803	0.047(0.000–1.401E4)	0.634		
pStage(I + II/III + IV)	2.243 (0.250–20.161)	0.471	1.478(0.330–6.615)	0.609		
CEA(≤5/>5)	0.045 (0.000–2.044E14)	0.602	0.046(0.000–3.688E4)	0.657		
CA125(≤35/>35)	1.000(0.000–2584.626)	1.000	0.046(0.000–4.070E7)	0.770		
CA199(≤39/>39)	0.039 (0.000–5.365E7)	0.762	0.042(0.000–1.509E4)	0.627		

*Abbreviations*: *OS* overall survival, *DFS* disease-free survival, *HR* hazard ratio, *CI* confidence interval, *RDW* red blood cell distribution width, *CEA* carcinoembryonic antigen, *CA125* carbohydrate antigen 125, *CA199* carbohydrate antigen 199

^a^Performed using the Kaplan–Meier analysis model and the log-rank test; values of *P* < 0.05 in the univariate analysis were entered into a multivariate analysis

^b^performed using Cox proportional hazards models with the forward likelihood method

## Discussion

Cancer is a leading cause of death in both more and less economically developed countries. In recent years, the fact that cancer may act as either a cause or a result of chronic inflammation aroused the attention to the connection between inflammation and malignancies [18]. Studies have demonstrated that the cancer-associated inflammation plays an important role in carcinogenesis and tumor progression [19, 20]. The possible mechanism may be that inflammation was associated with malnutrition, immune dysfunction, platelet activation, angiogenesis and activation of cytokines [21, 22]. Although the general factors of GC including tumor stage, lymph node metastases, and lymphatic vessel invasion have been used clinically for patient risk-stratification and the guidance of therapeutic strategy, the complexity of clinical conditions of cancer patients prompts us to search for more appropriate biomarkers to evaluate the patient’s general condition for therapeutic and prognostic purposes. Despite scientific efforts, there are few suitable serum/plasma biomarkers of GC, which have high sensitivity and specificity for the screening or surveillance of this malignancy.

Previous studies have demonstrated that hematologic parameters including NLR, PLR, MPV, RDW were significantly correlated with many malignant tumors, such as colorectal cancer, prostate cancer, esophageal cancer and breast cancer [23–26]. Therefore, in the present study, we detected whether the preoperative RDW and PDW value could distinguish the patients of GC and early GC from the healthy controls. In addition, we analyzed the association of RDW, PDW with the clinicopathological features of patients with GC. Furthermore, we evaluated the predictive effect of RDW and PDW on progression and prognosis of GC.

RDW, which is a measure of heterogeneity in erythrocyte size, is a sensitive and specific indicator of iron deficiency anaemia. Recently, there has been growing evidence that high RDW, frequently associated with inflammation and oxidative stress, increases overall and disease-specific mortality in patients with chronic or progressive inflammation diseases [10, 11, 27, 28]. Many studies have shown that it was closely related to the other inflammation markers such as CRP, IL-6 and TNF-α [27, 29]. It was also shown that RDW increased in the inflammatory intestinal disease in which chronic and active inflammation increased [10]. Emerging evidence has suggested that RDW might can be used as a marker for diagnosis or prognosis in various solid cancers. Moreover, most studies focused on RDW as an independent predictor of cancer survival. Seretis et al. [12] showed that RDW was significantly higher in patients with breast cancer compared to the patients with fibroadenomas and had a high correlation with the size of primary tumor, the number of metastatic axillary lymph glands and overexpression of HER2. Warwick et al. [30] found that preoperative RDW in patients undergoing pulmonary resections for non-small-cell lung cancer could predict mortality and long-term survival. Potential mechanisms of elevated RDW in cancer patients may be that carcinogenesis is usually accompanied by increased inflammation, which causes inhibited response to erythropoietin, reduced iron release from reticuloendothelial macrophages, and shortened red blood cell survival through relevant inflammatory markers. In addition, RDW was found to be associated with malnutrition, which has been shown to be correlated to lower response to treatment, poorer prognosis and quality of life [31, 32].

The first association between hypercoagulability and malignancy traces back to the observations made by Trousseau in 1865, yet today thromboembolism is one the most common causes of death in cancer patients [33]. Activated platelet leads to hypercoagulability. Platelet can be activated by inflammatory factors such as interleukin-6, and elevated platelet in peripheral blood is associated to the development of many cancers [34–36]. And that the induction of platelet synthesis leads to not only the change of platelet count but also function and physiology [15]. PDW is a direct flow cytometric measurement of platelet cell volume and clinicians pay less attention than platelet count. PDW seems to be a more specific indicator of platelet activation than mean platelet volume (MPV), since it was not elevated during single platelet distention caused by platelet swelling [37]. As we mention above, the systemic inflammatory response is associated with coagulation processes, although the precise mechanisms that underlie this response, as well as the interaction between coagulation, inflammation, and carcinogenesis remain obscure.

Through the comparison between groups, we found that the values of RDW in patients with GC were significantly higher than those in healthy controls and the PDW values in GC were significantly lower than those in controls. In addition to, in order to validate whether the RDW and PDW can become the differential diagnosis indicators of early GC, we collected 164 patients with early GC to compare with the healthy controls, finding that the differences were also significant. The results enlightened us that the RDW and PDW can be used as screening indicators of GC. Wang FM et al. [38] reported that the RDW values were significantly higher in patients with renal cell carcinoma than those with controls, which was consistent with our result. However for PDW,although several clinical studies have demonstrated that PDW was elevated in patients with chronic inflammatory and various carcinomas [14, 39], other studies have support our findings. Chronic inflammatory process in patients with inflammatory bowel diseases (IBD) results in increasing in the number of blood platelets and changing in their activation and morphological parameters, and PDW was significantly lower in active phase of Ulcerative colitis and Crohn’s disease groups than remission phase [40]. Kurtoglu et al. [41] found that decreased PDW values were significantly associated with endometrium cancer. This suggests that the reduction of PDW may be related to the progression or activation of disease, rather than simply related to a disease.

We investigated the relationships between RDW, PDW and cliniclpathological features and detected that a higher RDW was significantly associated with advanced disease such as older age, a larger tumor diameter, deeper tumor infiltration, and Lymph node metastasis while a lower PDW was significantly associated with advanced condition such as male, older age, a larger tumor diameter, deeper tumor infiltration, elevated CEA and CA125. The results above indicated that increased RDW and decreased PDW can be used as indicators of malignant progression in GC.

We carried out the follow-up and analyzed the survival rates in patients with GC and early GC. A high preoperative RDW predicted respectively worse OS and poorer DFS in patients with GC and early GC while a decreased PDW group only exhibited a poor DFS than low group in early GC. The results testified that RDW and PDW could be used to estimate the survival rates of the patients with GC. Furthermore, we conducted univariate and multivariate analysis to predict the independent factors of GC. A high RDW was significantly associated with poor DFS and had a borderline relationship with OS in univariate analysis. Although high RDW lost its independent prognostic significance for OS and DFS in multivariate analysis, it still offered considerable information on RDW for clinical prognosis. In addition, increased RDW or decreased PDW was significantly connected with worse DFS in univariate and multivariate analysis, which indicated that the RDW and PDW could be used as independent prognostic indicators of early stage GC.

An assessment of RDW and PDW levels to predict clinical outcomes in patients with GC has advantages. It can be acquired immediately when the patient is suspected of GC to assess the patient’s general condition objectively such as malnutrition and abnormal coagulation state, contribute to diagnosis and prognosis evaluation. However, several limitations need to be noted in this study. First, the major limitation of the present study is the determination of the cutoff values. The current literatures confirmed the optimal cutoff values for RDW and PDW according to ROC curves, median value or based on previous studies. We also analyzed the survival for GC using the cutoff values according to ROC and previous studied, but no correspondingly significant difference was detected. Second, the sample size was small,which may reflect a selection bias and be less persuasive; Third, our study was a retrospective study, so there may be potential bias and inaccuracy in data collection as in most retrospectively designed studies.

## Conclusions

In conclusion, as available and convenient biomarkers, the RDW and PDW have diagnostic power and can discriminate patients with GC and early stage GC from the healthy controls. In addition, the RDW and PDW can be used as significant indicators for progression and prognosis of GC. But it must be noted that measurement of single biomarkers identified far lack sufficient sensitivity and specificity may not always accurately provide information alone, which may be affected by many factors. It is likely that multiple markers like RDW and PDW will need to be employed simultaneously. Nevertheless, there is a need for prospective studies to understand the mechanisms underlying the alterations of RDW and PDW in carcinogenesis and chronic inflammatory conditions.

## Abbreviations

CA199: Carbohydrate antigen 199
CBC: Complete blood count
CEA: Carcinoembryonic antigen
DFS: Disease-free survival
GC: Gastric cancer
IBD: Inflammatory bowel diseases
OS: Overall survival
PDW: Platelet distribution width
RDW: Red distribution width
